# The importance of developing a financial audit process: An academic medical center perspective

**DOI:** 10.1017/cts.2023.650

**Published:** 2023-10-13

**Authors:** Jessica Fritter, Cody Harper

**Affiliations:** 1 The Ohio State University College of Nursing, Columbus, OH, USA; 2 Nationwide Children’s Hospital, Columbus, OH, USA

**Keywords:** Clinical research business operations, feasibility, financial audit process, clinical trial operations, clinical trial management systems

## Abstract

Clinical research studies run the risk of being in a deficit leading to premature study termination or a desperate struggle to find new funding to continue the research. It is important for institutions, small or large, to have financial oversight during the research process. We created a financial audit process for a core clinical research department at a pediatric hospital. Understanding where to find your costs, what costs are important, and other elements of the audit process are essential. Knowing how to replicate a financial audit process can help you eliminate the risk of a financial deficit.

## Introduction/Problem

Clinical trial budgeting takes finesse and great attention to detail. There are multiple components in the clinical trial budget process. The budget is a critical piece to knowing if the trial is feasible and profitable. The components to consider when designing a trial budget are type of study, volume of subjects to be enrolled, personnel costs, site costs, procedures, startup, and close-out fees, among many others [1]. As trials have become more complex, there are additional costs to consider, including amendment fees, secondary regulatory fees, monitor change fees, adverse event costs, and pharmacy costs, among many others. It is essential to think of all costs associated with the trial and protocol in question. During the budget construction process, there are questions to be answered and tools to consider. There is benefit in asking and finding out the number of patients and sites included in the trial, per-patient costs, personnel needed and their payment structure, schedule of events, and gaining input from the institution, principal investigators, and coordinators [1]. Analyzing these when creating the budget is crucial because it can set the trial up for success. However, something to consider is how to monitor the budget during the trial. Monitoring the budget throughout the trial can create financial stability throughout the trial’s longevity. We will be providing insight on one way to track the financial status of your trial and that is through financial audits.

## Financial Oversight

It is important to conduct financial audits because it provides a comparison of your total revenue to your total expenses. There is the ability to notice the total hours staff is working on the trial versus what was budgeted and if the trial is over or under budget regarding labor. This allows research administration to analyze if staffing models need to shift [2].

When assessing where to find the costs to audit, there are multiple places to look to capture those costs. First, ask the sponsor for the final proposed budget that includes their starting costs, then review the protocol and schedule of events to help establish the costs for the trial, next review with the principal investigator and research staff to identify historical internal costs, and finally talk with accounting or the sponsored projects office to identify any charge masters with costs [2]. Another key aspect of financial oversight is understanding the studies' post-launch billing cycle. This is reviewing the contract and budget, feasibility assessment, and managing monthly finances all in relation to the investigator’s research enterprise and the department’s research enterprise [3]. The contract and budget hold the formal agreement of the payment terms and payment milestones, the feasibility assessment shows the sites' expenses in comparison to the sponsors' payment structure, and when managing the monthly finances, this is the process of tracking invoices and payments to ensure accurate accounting. These three elements are key in analyzing the investigators and department’s financial stability. From the investigator’s perspective, there needs to be monitoring of the financial progress and total revenue versus total expenses. This should be captured in real time by the study staff because if there is a delay in recording the charges there could be a result in a study deficit. For the departments' infrastructure, there is a need for a strong financial system to analyze the strengths and weaknesses of the research infrastructure [3]. All three elements must also be analyzed with a time element in play as new budget versions come along and some items may invoice for lower costs.

## Elements of a Financial Audit

### What Are You Looking For?

It is impossible to tell what money is missing without knowing what is available. When developing the audit process, we identified 3 major categories needed for a successful audit. First are startup costs, including IRB fees as well as ancillary services fees such as pharmacy and regulatory procedures, among many others. Second, is any invoiceable events that can be billed out during the study functioning. This would include events like data entering, monitor visits, amendment fees, and coordinator calls. We found that these are the most missed data points and can quickly total up to thousands of dollars missed per study per year. Anecdotally, in the first year of implementing our audit process, we found close to $300,000 dollars in missed charges mainly due to missing data. Third is the budget and calendar itself, this is for the study visits and is all-encompassing. At our institution, we capture study payments through our clinical trial management system, and depending on the study some may be auto-paid, and others need to be manually submitted. Typically, the first two parts are included in this category, but they were listed first as they fall to the fringe of the calendar visits themselves.

Copies of these should be available through your grants/sponsored projects office or regulatory staff assigned to work on the given study. In the case of the institution where we implemented this, it is contained in an electronic storage area as either PDF or Excel sheet. This may differ at other institutions where their preferred method of storage is in a physical binder or in a physical study folder. When acquiring the documents, it is worthwhile to find an event log or schedule of events for the study to see what is eligible to be or has already been invoiced. If these are scattered and in multiple formats, it may prompt the need to develop a better storage format for future audits.

### Electronic Systems

**Figure 1.**
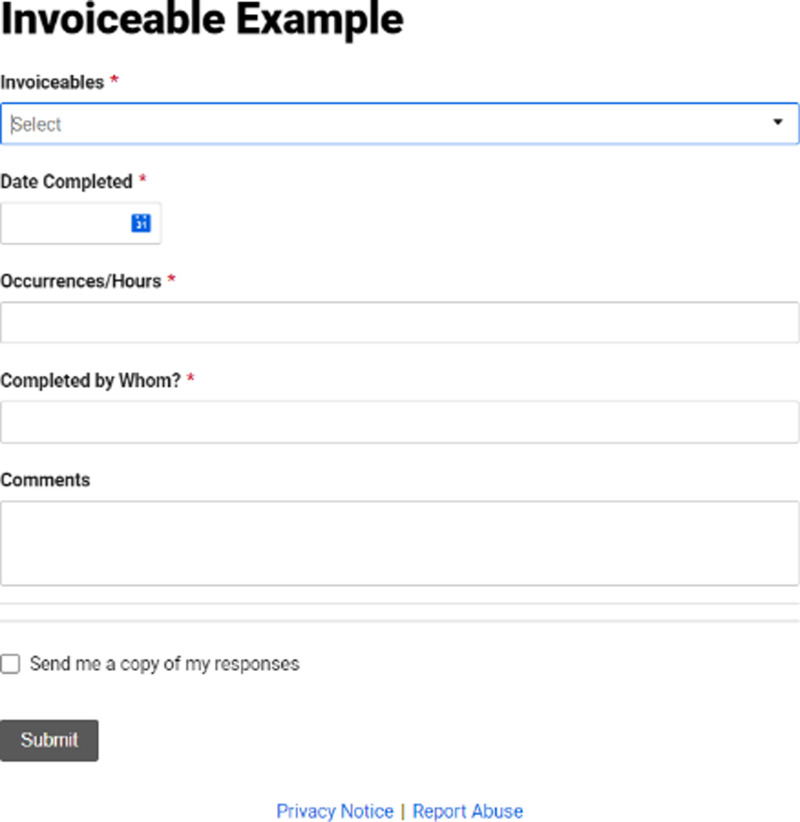
Invoiceable automated form.

Electronic storage systems or financial tracking systems are the most efficient way to maintain data integrity throughout a clinical research study. This could be something as simple as Excel with a two-column list containing every financial event and dollar amount. However, it can grow in complexity to software such as OnCore or Smartsheet that allows for monthly reminders and forms for staff to log the events. Then this could lead to an automated monthly invoice going out that can be verified with the coordinator reducing the odds of billing errors between lengthy audit gaps. Fig. 1 below shows what this automated form looks like at our institution.

**Figure 2.**
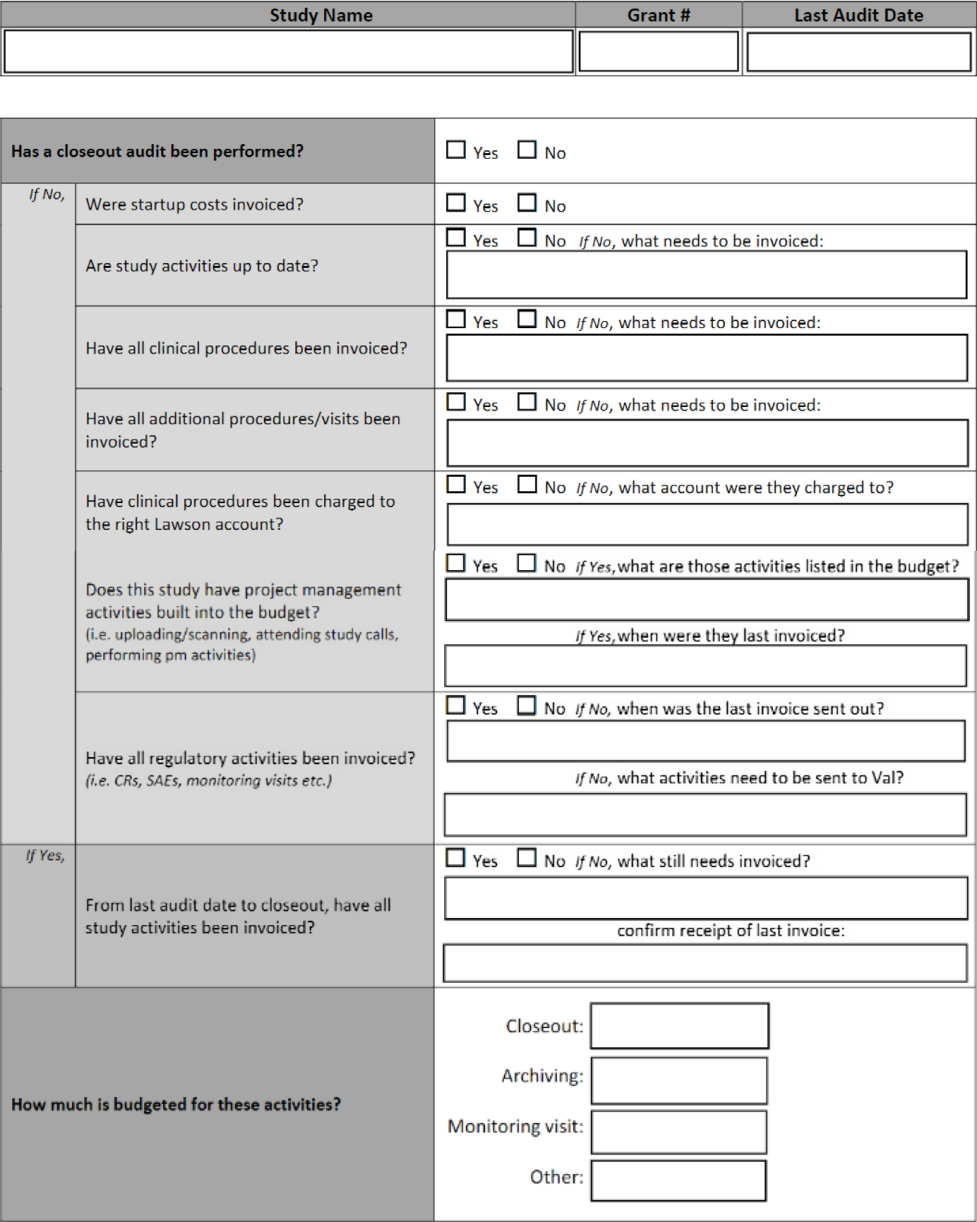
Study audit form.

During the audit logs or Excel sheets will be compared to find what events occurred but no invoice was sent. Either in the Excel sheet or the software of choice, it is recommended to log all audit findings for the given study. At our institution, a form shown in Fig. 2 shows how the audit was logged and then saved in a software like Smartsheet. This can be compared throughout the study lifecycle to fix problem areas and compare studies to recognize where the department may be seeing the most financial deficits. Software can also be used to assign tasks with reminders for the given audit as well as schedule the next audit. To reiterate, this can be done in an Excel sheet stored in a shared drive, but more complex software is available to help automate the process.

The software used in the development of our process was OnCore for event logging and invoice generation, Smartsheet for audit history trackers, and eTrac plus 2 former systems for study event space. It is important that any software utilized is ALCOA compliant and follows the guidelines of the local IRB.

### Financial Audit Process

Before the studies audit meeting, it is important to note that in the 3 years of implementing the financial audit process, we have seen a drastic decrease in missed funding findings. The first year it was implemented led to over $280,000 in findings and only $51,000 the following. This year the department is projected to have only $26,000 in findings. All 3 years are including old studies closing out and new studies coming in.

Each month there is a financial audit meeting. For these meetings, a variety of people are required to make sure no money is left on the table. A regulatory staff member is needed to verify the regulatory line items, such as amendments, were invoiced. A clinical staff member will ensure visits were properly logged in the OnCore system to ensure adequate invoicing will occur through that automated system. A system admin will check the electronic system and record everything accurately. A financial administrator will analyze the study balances and forecast its potential revenue or loss of revenue.

During this meeting, we review a set of studies being conducted through the core. The number of studies in a given meeting and number of meetings in a calendar year may vary based on the number of core studies available to audit. The studies selected are long-running studies prioritizing those that are closing or recently closed or studies that have not been audited in the past calendar year. This schedule is laid out by December at the latest for the following year’s audits. Throughout the process, a study audit form is filled out outlining all the major areas listed above and the financial administrator will submit the study audit form. See Fig. 2 below for the study audit form sample.

## Conclusion

Understanding the financial process of a clinical trial is important to the success of the trial. There are resources out there to set up feasibility assessments and ensure a strong budget is constructed prior to the start of the trial, however, very little is out there on conducting financial audits for clinical trials. The audit process consists of shared understanding of the budget pre-award, building the calendar and invoice terms in the clinical trial management system, scheduling audit meetings post-award, and a follow-through of the audit process. Following the audit process outlined in this manuscript has proven to return hundreds of thousands of dollars in revenue for our institution. Most importantly, educating your research staff on what data points need to be captured for financial profitability in the study is key, but also sharing with other departments on the audit process is valuable. This is a significant component of ensuring sustainability, profitability, and knowing when budget renegotiations need to take place. Clinical trials are groundbreaking and for them to continue, there needs to be adequate budgeting.
